# Rational antibiotic prescribing in primary care: qualitative study of opportunities and obstacles

**DOI:** 10.3399/bjgpopen20X101079

**Published:** 2020-09-30

**Authors:** Pär-Daniel Sundvall, Ingmarie Skoglund, Maria Hess-Wargbaner, Christina Åhrén

**Affiliations:** 1 Region Västra Götaland, Research and Development Primary Health Care, Research and Development Centre Södra Älvsborg, Borås, Sweden; 2 Primary Health Care, Department of Public Health and Community Medicine, Institute of Medicine, The Sahlgrenska Academy, University of Gothenburg, Gothenburg, Sweden; 3 Region Västra Götaland, Swedish Strategic Program Against Antimicrobial Resistance (Strama), Gothenburg, Sweden; 4 Centre for Antibiotic Resistance Research (CARe), University of Gothenburg, Gothenburg, Sweden; 5 Department of Infectious Diseases, Institute of Biomedicine, The Sahlgrenska Academy, University of Gothenburg, Gothenburg, Sweden

**Keywords:** Antimicrobial stewardship, primary health care, healthcare team, health care quality, antimicrobial drug resistance, qualitative research

## Abstract

**Background:**

The Swedish strategic programme against antibiotic resistance (Strama) has worked towards rational use of antibiotics, and Swedish antibiotic prescribing is low.

**Aim:**

To explore how opportunities and obstacles for rational antibiotic prescribing were perceived by primary health care centres (PHCCs).

**Design & setting:**

A qualitative study of 50 randomly selected reports from approximately 200 PHCCs in 2013 and 2016 in Region Västra Götaland, Sweden.

**Method:**

One assigned GP at each PHCC reported yearly in an open-ended questionnaire on how the PHCC worked to improve antibiotic prescribing. The report included several antibiotic-related tasks and a summary of reflective meetings with the doctors, the head of the PHCC, and, preferably, also the nurses. The reports were qualitatively analysed using Malterud’s systematic text condensation (STC).

**Results:**

*'Everyone wants to do right, but sometimes you do not know what's right or wrong.*' Knowledge about diagnosis and treatment of infectious diseases was highlighted. Knowledge and skills had to be internalised by the clinician in order to bring about behavioural change. This could be achieved through reflective, collegial dialogues where consensus often was found. Structural factors at the PHCC could provide good conditions for '*doing right*', but could also constitute obstacles. Teamwork involving all personnel was important to achieve rational antibiotic prescribing.

**Conclusion:**

Enablers for rational antibiotic prescribing were knowledge, reflective collegial dialogues, a well organised workplace, and a collaborating team. Obstacles were lack of knowledge, insufficient staffing, perceived lack of time, and overuse of laboratory tests. Patients’ attitudes and expectations could be both.

## How this fits in

The rational use of antibiotics, as well as improving antimicrobial stewardship programmes, is an international priority. Knowledge itself is not enough to change antibiotic prescribing behaviour. This research contributes to the understanding of how knowledge and skills can be internalised by the clinician through reflective collegial dialogues, resulting in change in the clinician’s behaviour. Teamwork that included all staff was also important in order to achieve rational antibiotic prescribing.

## Introduction

The rapid spread of antibiotic resistance is a huge problem as modern health care depends on effective antibiotics to treat bacterial infections.^1^ Therefore, a rational use of antibiotics, as well as improving antimicrobial stewardship programmes (ASP), is an international priority. The effect of antimicrobial resistance policies seems to be variable, and their effectiveness in controlling antimicrobial resistance is not yet fully understood.^2^ Policies encouraging responsible use of antimicrobials in primary care have been proven to be effective, but are not easily generalisable.^2^ Audits and feedback improve antibiotic use, and are core components of ASPs, as passive educational materials are not enough.^3^ However, if audits and feedback are removed, the initial benefits may be lost; therefore, ASPs require continued active efforts.^4^ In a qualitative study exploring experts' attitudes on strategies to promote rational antibiotic use, the experts underlined the importance of providing flexible interventions engaging GPs, and addressing GPs' concerns about recommendations.^5^

The Swedish strategic programme against antibiotic resistance (Strama) has worked towards rational use of antibiotics, and Swedish antibiotic prescribing is low.^6^ The decrease in prescribing has, in recent years, been larger in Region Västra Götaland (VG) compared to most other Swedish regions.^7^ The multifaceted ASP, carried out by Strama VG in primary health care, has included components not earlier described in the literature. As further research is needed to identify the essential actions for an effective and versatile intervention,^8^ it is important to study the experiences of the participants in the ASP carried out by Strama VG. The aim of this study was to explore how opportunities and obstacles for rational antibiotic prescribing were perceived by the PHCCs in VG.

## Method

### Participants

Since 2012, Strama VG has had a ‘contact GP’ in each of the approximately 200 PHCCs in VG, as part of the ASP in primary care. These contact GPs were educated to lead reflective collegial meetings, and received a package of interventions to encourage and facilitate rational use of antibiotics. Respiratory and urinary tract infections, as well as skin and soft tissue infections, are the most common infections treated with antibiotics in Swedish primary care.^7^

### Data collection

Each year (2013 to 2016), the contact GPs wrote a report on their work to improve antibiotic prescribing at the PHCC, including conclusions after a mandatory reflective dialogue meeting with all doctors, the head of the PHCC, and, preferably, also nurses. The items conducted prior to or during the meeting were:

- discussion of case studies with didactic questions and tutor manuals provided by Strama VG, led by the contact GP;
- reflection on antibiotic prescribing, for each individual GP, including locum physicians;
- scrutinising each other’s medical records on infection treatment versus guidelines;
- doing a 20-question quiz or web-based e-learning on treatment guidelines;
- educational session with personnel at the PHCC using PowerPoints with guided lecture notes provided by Strama VG.

The work included mandatory (I to III) and voluntary (one of IV or V) parts. The report was based on open-ended questions on their conclusions from their yearly work, improvements undertaken, plans for future actions to improve prescribing behaviours or desires for actions from Strama VG, and the GPs' thoughts about the reflective meetings and the report. The report was reimbursed if completed, and was preceded by reflective collegial dialogue meeting(s) including the items conducted prior to or during the meeting, as described above. The reimbursement depended on the size of the PHCC, and varied between 32 000 to 70 000 SEK in 2013, and 13 000 to 32 000 SEK in 2016. The reimbursement was paid regardless of success or not regarding perceived rational antibiotic prescribing, or whether there was description or discussion of weaknesses and areas for possible improvements.

The reports from 2013 and 2016 were qualitatively analysed, with the aim of capturing perceptions from as different a period as possible.^5^ In total, *n* = 186/201 PHCCs completed the report in 2013, and *n* = 163/199 in 2016. These reports were randomly arranged for each year using Microsoft Excel, and then read in this order. After having read 25 from each year, no new items were perceived.

### Qualitative approach, research paradigm, and analysis process

Malterud’s STC was used in the analysis.^9^ The method is inspired by Giorgi’s phenomenological analysis^10^ and included the following steps;

1. two authors (PDS and IMS) read the material several times, initially independently, to obtain an overall impression of the data;
2. the authors identified units of meaning representing different aspects of the research question, and performed coding and sub-coding of these;
3. the contents of each of the coded groups were condensed and summarised;
4. descriptions were developed, reflecting the informants’ most important experiences of perceived opportunities and obstacles for the achievement of rational antibiotic prescribing.

STC offers a process of feasibility, intersubjectivity, and reflexivity during the analysis of qualitative data. The methodological rigour makes the steps easy to follow.

Three of the researchers had experience of working in primary health care (PDS, IMS, and MH), and three had experience of working with Strama (PDS, MH, and CÅ), and the level of prior understanding of the context was high, except for IMS. To achieve trustworthiness, the researchers mainly responsible for the text analysis were engaged in an ongoing discussion and reflection throughout the analysis. Discussion and dialogue were also held with the two other authors.

## Results

Knowledge and skills in different forms were highlighted. The emerging concepts were logical knowledge; practical and technical craftsmanship, including logistics and organisation; and wisdom and reflective collegial dialogues.

### Logical knowledge

The importance of regular education and lectures on treatment guidelines for the diagnosis and treatment of infections was highlighted by many. This was true for doctors, nurses, and other staff at PHCCs, including child health care centres, nursing homes, and home care. The Centor’s criteria for pharyngotonsillitis were not known in some workplaces, even in 2016. Uncertainty about the guidelines might lead to increased antibiotic prescribing. Scepticism towards applying Strama’s mindset was expressed by a few:

*'Everyone wants to do right, but sometimes you do not know what's right or wrong.'*

*'Involve ALL staff in education to ensure that patients at all levels get adequate expectations regarding antibiotics and their use.'*

A folder on national updated treatment guidelines on common infections in primary care (published by Strama, the Public Health Agency of Sweden, and the Swedish Medical Products Agency of Sweden),^11^ as well as other Strama information was highly appreciated. To serve as a knowledge support, the materials had to be in place when needed.

In case of urinary tract infections in the older patients, insufficient knowledge of current treatment guidelines, and too frequent a use of urine dipsticks might lead to far too many prescriptions, and the assistant nurse's knowledge seemed to need improvement.

Knowledge could be confirmed by case studies and quizzes from Strama:

*'The answers* [to case studies with didactic questions/Strama quiz] *are now perceived as self-evident, and we are well aware of current guidelines for treatment.'*

### Practical and technical craftsmanship, logistics, and organisation

Good organisation for taking care of all kind of infections was important. Participants emphasised the importance of having enough time and not making assessments too quickly.

### Staffing

Many experienced that having a stable team of medical staff, with good accessibility and continuity, promoted rational use of antibiotics. Insufficient staffing negatively influenced prescribing. High staff turnover made it difficult to maintain rational antibiotic use. Prescribing patterns that were not compliant with guidelines were described among locum physicians, especially those who were not specialised in general practice. Additionally, following locum physicians’ prescribing statistics was technically difficult. Some GPs considered introducing scheduled education sessions with future locum physicians, and some had already begun with this:

*'For the past 1.5 years, we have had a larger proportion of temporary staff compared to earlier, often in short temporary positions, which made it harder to implement new guidelines throughout the staff.'*

*'A great improvement is that we only have long-term 'locum physicians' now, who attend our meetings and internal education.'*

### Revisits, drop-in surgery, and lack of time

Time pressure could increase antibiotic prescribing and there were difficulties in offering re-visit appointments. A free return visit within a week could reduce prescribing:

*'The poor general availability in primary care is likely to increase antibiotic prescribing, as it is not always possible for the patients to get an appointment if they get worse.'*

There were two sides of the coin; easily accessible drop-in surgery might result in too many prescriptions. This risk could, however, be overcome by well-developed routines and good cooperation among personnel. Also, patients could easily return, and antibiotics need not to be prescribed for safety’s sake:

*'The increase* [in prescriptions] *can be explained by our high availability with drop-in surgery for emergency visits...'*

*'We have a drop-in reception at our PHCC, which allows us to easily assess the patient, decide to wait for treatment, and to offer re-visits/follow-ups liberally.'*

### Nurses and the team

The nurses’ triaging of patients seeing the doctors was emphasised, and checklists were considered to be a good tool for a uniform triage. Regularly talking with each other, and providing gentle reminders could help maintain a good way of working. With sufficient self-care advice, the patient usually waits before seeking care:

*'The nurses assess some uncertain cases themselves before booking doctor visits, but it would be desirable that they could see more patients to assess if they are 'sick' enough. The fewer patients who see a doctor, the less risk they will get an antibiotic prescription.'*

In wound care, the doctor was often involved at a late stage, which is why the need for cross-professional teams was highlighted:

*'Here you probably have to resist demands for* [bacterial] *cultures and treatments to a greater extent, which goes well if you have seen the patient at the PHCC or the nursing home. However, this will be harder if it is the typical Friday afternoon telephone consultation with the district nurse.'*

### Clinical examination and laboratory tests

Several GPs estimated that C-reactive protein (CRP) was used too frequently, and should not be used when the general condition and vital parameters were unaffected:

*'*[Regarding] *acute tonsillitis, the Centor-criteria are generally well followed. However,* [we] *frequently note cases where there were negative rapid antigen detection tests, and yet treatment* [was provided]*, not rarely because CRP was taken for safety's sake, and then the CRP concentration was elevated and therefore treatment was initiated, because of the increased CRP concentration.'*

Many informants considered rapid antigen detection tests (RADT) for beta-haemolytic streptococci group A (GAS) to be taken too often before clinical assessment of pharyngotonsillitis, especially when coughing was present. On the other hand, it was emphasised that RADT for GAS should be taken more frequently before actually prescribing antibiotics for pharyngotonsillitis. Some stated that well-trained nurses may decide to use a RADT for GAS in patients with three or more positive Centor criteria indicating pharyngotonsillitis:

*'The group of GPs therefore decided that, hereafter, RADT for GAS cannot be performed on a patient without prior medical examination. This decision has been raised at a workplace meeting so that all healthcare professionals receive the same information.'*

Many informants documented the general condition and vital signs, such as respiratory rate and pulse oximetry, before making decisions.

Some further success factors were liberally culturing for bacteria prior to antibiotic treatment, and more frequent use of computer tomography of the sinuses when suspecting sinusitis, tympanometry, and chlamydia tests, etc.

Enough time for current medical history and assessment was needed; these were often more conclusive than laboratory tests, and should precede sampling. The need for antibiotics should be questioned, and refraining from prescribing was often necessary in medically unaffected patients. Instead, symptomatic treatment was emphasised. In well-chosen cases, it might be appropriate to deviate from the treatment guidelines:

*'Not to start with sampling* [before medical assessment] *is a rule that needs to be implemented at the PHCC, so that everyone does the same thing, simply.'*

### Patients

Patients' attitudes and expectations of receiving antibiotics might affect prescribing. Many described that over time, patients, to a greater extent, wanted to avoid antibiotics. It was time-consuming to inform patients about pros and cons of antibiotic treatments, and the normal time course of infections, especially if the patient was worried about complications when refraining from antibiotics. Written information summarising treatment guidelines, posters, and information leaflets in waiting rooms were effective and appreciated:

*'We also put a lot of effort into explaining to the patient why they do not get antibiotics, as this sometimes might be a source of irritation.'*

Frequent attenders and those accustomed to liberal antibiotic usage were risk groups for getting more antibiotics:

*'Some patients have different antibiotics at home* [bought from their home country]*, and they start using the medicine before they come to the visit.'*

High morbidity among the population and nursing homes was perceived as resulting in higher prescribing. Seasonal variation in infection rate, number of patients during the vacation period, and the closure of nearby PHCCs were highlighted:

*'* *... the PHCC has expanded considerably and has suddenly gotten significantly more patients ... which means we might have become less strict with criteria for treatment due to increased stress. The newcomers also have different treatment-seeking patterns ... with higher expectations of getting treatment right away ... they have longer to drive ... which makes them less likely to return after deterioration, and then they rather want the doctor to make a prescription right away.'*

### Wisdom and reflective collegial dialogues

The GPs' reflective collegial dialogue was highlighted as they received practical tips from each other, and this was important in achieving rational antibiotic use. Continuous reconciliation helped to develop health care at the PHCC. Knowledge and skills had to be internalised by the clinician in order to bring about behavioural change:

*' … but in order to achieve change, it is important to have a collegial group where you can safely discuss your mistakes and shortcomings … '*

*' ... each quarter we discussed and commented on prescription data* [received quarterly from Strama VG]*, and reflected on why they look like they do ... it has also made us constantly active in the discussion and aware of our own prescriptions.'*

These dialogues were particularly important for specialist trainees and locum physicians, leading to repeating the memorandums. Indications for antibiotic treatment were sharpened and adherence to guidelines increased. Several stressed the assiduous daily work that included continuous supervision of interns and specialist trainees. Improvements were noted without the number of mortalities increasing:

*'Nothing special but we are very conscientious about providing regular Strama-updates, especially for the doctors.'*

*'We consider the decreasing curve* [for antibiotic prescriptions] *to be the fruit of our ongoing discussion of prescription patterns, policies, and routines within the GP team. We have followed the region's prescription rates, and we have monitored our own prescribing rates, and we have managed relatively well in our ambition.'*

Consensus often was reached on the treatment of infections during the reflective collegial dialogues. By consensus, the prescribing was sometimes optimally low, sometimes evenly spread, but could be 'wrong' or high. Several noted differences between younger and older colleagues, presumably dependent on how to adapt to new guidelines and the use of new methods. Different cultures and traditions were also seen in the broadest sense. Others found significant differences in number of prescriptions and choice of antibiotics, especially in acute bronchitis:

*'We need to continue to imprint on everyone the idea that doxycycline is not a cough medicine.'*

The importance of a good climate where mistakes and deficiencies could be openly discussed was emphasised. Daring to challenge one another resulted in compelling discussions:

*'There was a lively discussion about the reason* [for the high prescribing rates]*: ... it must be temporary doctors ... , not me, there must be someone else*.'

Regularly consulting with the colleagues at the PHCC was a success factor, as well as regular tutorial of specialist trainees and interns in primary care. Nurses involved in telephone counselling, physicians, and all other staff members needed to have uniform strategies in all patient contacts.

Working with the yearly report to Strama was generally considered positive and the structure provided opportunities for yearly collegial learning with reflective meetings. The work could encourage the head of the PHCC to review local routines and improvement areas:

*'The discussions were good; there was a learning atmosphere in the environment.'*

The fictitious case studies provided by Strama, accompanied by didactic questions and tutor manuals, were considered meaningful and instructive, and gave close-to-realistic opportunities to reflect around their practice. There were no major problems in reaching consensus among colleagues when discussing the case studies. Strama’s prepared PowerPoint presentations, including lecture notes, were useful for internal training at the PHCC, and the quizzes together with joint discussions were well suited for internal team education. Scrutinising each other’s medical records regarding treatment of infections and comparing this with guidelines were an appreciated part of the reflective meetings. However, some expressed that the amount of medical records to scrutinise (three per doctor) was too many:

*'The Strama project has a good foundation in reality and everyday life, and does not feel imposed at all, but natural. However, I think that the self-declarations* [the yearly reports] *should not be made more extensive than this.'*

A few informants expressed that the yearly work was too extensive and stressful:

*'I have probably mentioned earlier that I never liked the self-declaration because I feel I am being very micromanaged and imposed upon. If you do not do just as you are told, the PHCC loses a lot of money. At the same time, I understand that the money forces a discussion about Strama issues that may be useful.'*

## Discussion

### Summary

Three concepts emerged when exploring how GPs perceived opportunities and obstacles for the achievement of rational antibiotic prescribing. 1) Logical knowledge denoted the informants’ perceptions that they wanted to do right, but it was not that easy. To succeed they needed education about how to diagnose and treat infections. 2) Practical and technical craftsmanship, including logistics and organisation, represented the informants’ expressed need for having enough time for patient consultation and not having to make assessments too quickly. Other structural factors, such as staffing and organisation, could provide good conditions for 'doing right', but could also constitute obstacles. 3) Wisdom and reflective collegial dialogues represented the process whereby knowledge and skills had to be internalised by the clinician in order to bring about behavioural change. This could be achieved through reflective collegial dialogues, where consensus was often found. Teamwork that included everyone, not only doctors, was emphasised, as well as time to reflect on one’s work.

### Strengths and limitations

This study has been conducted by researchers with experiences from both the quantitative and the qualitative spheres of knowledge. Throughout the process of analysis the findings were discussed, as there is no one single 'truth' that can be validated in the interpretation.^12^ Other colleagues experienced in qualitative and educational research were also engaged. The use of open-ended reflections from the contact GPs on what measures need to be done concerning ASPs is unique, and the random ordering of the PHCCs chosen is also a strength. However, there might have been some selectivity in the reporting of obstacles and barriers as the annual report covered pre-selected topics. The results in this study are consistent with a previous study showing that several factors are important for rational antibiotic prescribing in primary care: the GP’s diagnostic process, internalised guidelines, nurse triage, patient expectation, and promoting management leadership.^13^ The authors’ previous knowledge of the area might be both a strength and a weakness. The fact that they have similar experiences from their practical work has enabled them to understand the written text in a richer way. On the other hand, persons with another background might have interpreted the texts slightly differently.

### Comparison with existing literature

#### Knowledge philosophy has evolved into system one and system two

The informants described different forms of knowledge. The nature of knowledge has been a philosophical concern throughout the centuries. Logical knowledge, or ‘knowing that’, corresponds to facts, understanding, and how to use knowledge in new situations.^14^ Practical and technical craftsmanship, including logistics and organisation, or ‘knowing how’, corresponds to skills developed through practice and exercise, and might be tacit. Wisdom and reflective collegial dialogues correspond best to the notion of familiarity, and in terms of medical practice, the notion of clinical intuition, gut feeling,^15^ and the recognition of holistic patterns.^16,17^

According to recent research in neuroscience and psychology, there are numerous indications that familiarity is built up in the memory system in a way that differs from the way learning has previously been described, and that this is exercised by two separate systems, the implicit system 1 (S1) and the explicit system 2 (S2).^18^

S1, oldest and subconscious, is characterised by recalling and recognising situations with automation, skills, abilities, and familiarity, and is often difficult to change, according to educational research. S2 develops in early childhood, based on reflection and verbalisation, including facts and understanding. Understanding is seen as a collaboration between S1 and S2.^19^ This knowledge can be applied concerning antibiotic treatment; it is not enough to focus on S2, but also on automated knowledge, as in S1.^19^ For low antibiotic prescribing it is crucial to have a common practice in the PHCC. Moreover, in low prescribing PHCCs, new knowledge was not only internalised in the PHCC as a whole, but also in all personnel through an ongoing professional discussion.^13^ This is in line with the results from the present study.

#### Communities of practice

Since the end of the 20^th^ century, learning has increasingly been viewed as a process involving social interaction, whereby community of practice (COP) is important for learning. Social anthropologist Jean Lave and teacher Etienne Wenger defined the concept as follows: 'communities of practice are groups of people who share a concern or a passion for something they do, and learn how to do it better as they interact regularly.'^19^ In the current context, the groups were practitioners having an interest in common. Learning occurred through contact with each other, such that social interaction facilitated perceiving information and knowledge acquisition. An example of learning in a COP way is how the yearly reports to Strama are organised; currently active professionals are given the opportunity to discuss relevant issues and to learn while doing so. To allow reflection as a part of education, it has been proposed that the following items are important: safety and confidentiality, time apart from daily life, and awareness of how the professional identity develops.^20^ In line with this study, a systematic review and meta-ethnography suggested that ASPs should incorporate five aspects to promote rational antibiotic use: allow GPs to reflect on their own prescribing; help decrease uncertainty about appropriate management of acute respiratory tract infections; educate GPs about appropriate prescribing; facilitate more patient-centred care; and be beneficial to implement in practice.^21^ COP might have similarities with social trust, as described in a Swedish government’s official investigation report.^22^ Soft measures, such as social norms, organisational culture, and leadership — in practice, concepts close to sociology and anthropology — are regarded as instrumental for gaining good results in an educational context, and these may also be applicable for optimising antimicrobial stewardship programmes.

#### Worldwide attitudes concerning use of antibiotics survey

Antibiotics are used worldwide. However, knowledge about appropriate use varies considerably, and this must be considered when meeting patients from different cultures.^23,24^ Following increasing migration, different patterns of knowledge may be encountered, both in professionals and in the care-seeking population. Several studies have shown that patient–doctor relationship and communication are important factors for antibiotic prescribing.^25^ The methods developed in the yearly reports to Strama seem to meet many of these different needs and the most recent knowledge known as COP.

### Implications for practice

The exploration of how GPs perceived opportunities and obstacles for the achievement of rational antibiotic prescribing revealed three emerging concepts: logical knowledge; practical, and technical craftsmanship, including logistics and organisation; and wisdom and reflective collegial dialogues. In modern times, such concepts are recognised as implicit S1, S2, and COP. Taken together, they may further understanding of memory and learning in the context of ASPs. Several items in the yearly report to Strama supported the notion that memory and learning were guided by these concepts. Opportunities consisted of having knowledge about how to diagnose and treat infections, having enough time for patient consultations, facilitating structural factors, and teamwork including all staff. Knowledge and skills had to be internalised by the clinician in order to bring about behavioural change. This could, in practice, be achieved through reflective collegial dialogues, where consensus was often found. Obstacles such as being uncertain about diagnostics and clinical treatment, lack of education, shortage of colleagues, working alone, time pressure, and insufficient time to reflect on one’s work associated with poor workplace organisation should be eliminated.
